# Point of Care Diagnostics for HIV in Resource Limited Settings: An Overview

**DOI:** 10.3390/medicina54010003

**Published:** 2018-03-13

**Authors:** Sello Lebohang Manoto, Masixole Lugongolo, Ureshnie Govender, Patience Mthunzi-Kufa

**Affiliations:** 1 National Laser Centre, Council for Scientific and Industrial Research, PO Box 395, Pretoria 0001, South Africa; MLugongolo@csir.co.za (M.L.); PMthunziKufa@csir.co.za (P.M.-K.); 2 College of Science, Engineering and Technology, Department of Physics, NB Pityana Building, University of South Africa, Science Campus, Florida 1710, South Africa; 3 The Innovation Hub, PO Box 1, Pretoria 0001, South Africa; ureshnie@gmail.com

**Keywords:** HIV, point of care (POC), resource limited settings

## Abstract

Human immunodeficiency virus (HIV) is a global health problem. Early diagnosis, rapid antiretroviral therapy (ART) initiation and monitoring of viral load are the key strategies for effective HIV management. Many people in resource limited settings where timely access to medical care is a challenge and healthcare infrastructure is poor have no access to laboratory facilities and diagnosis is dependent on the presence of point of care (POC) devices. POC instruments have shown to be easy to operate, maintain and transport and can easily be operated by less skilled health workers. Additionally, POC tests do not require laboratory technicians to operate. POC devices have resulted in a growing number of people testing for HIV and thereby receiving treatment early. In recent years, there has been great improvement in the development of POC technologies for early HIV diagnosis, HIV viral load and cluster of differentiation 4 (CD4) measurement. This review discusses POC technologies that are currently available and in the pipeline for diagnosing and monitoring HIV. We also give an overview of the technical and commercialization challenges in POC diagnostics for HIV.

## 1. Introduction

The human immunodeficiency virus type 1 (HIV-1) is responsible for causing acquired immune deficiency syndrome (AIDS) and was first recognised in 1983 [1]. The devastating effect of HIV infection is known throughout the world and can be considered among the greatest pandemics in human history. Globally, at the end of 2014, an estimated 36.9 million (34.3 to 41.4 million) people were living with HIV and 2 million (1.9 to 2.2 million) people became newly infected with HIV (UNAIDS). As from June 2015, 15.8 million people infected with HIV had access antiretroviral therapy (ART), compared to 13.6 million in June 2014, and 1.2 million people died from AIDS related illnesses at the end of 2014 [2]. Sub-Saharan Africa is the most widely affected region, with approximately 70% of HIV all infection. New infections of HIV have declined by 35% globally since 2000 and this can be attributed to the massive progress in diagnostics and treatment. In order for the United States 2020 National HIV/AIDS Strategy to be achieved, a massive increase in the HIV testing capacity is required [3].

Testing for HIV plays an essential role in the disease control and prevention because knowledge of the patient’s status is beneficial to the individual and for public health. Accurate and early HIV detection is of essence as HIV transmissibility and infectiousness are high during early infection [4]. If infected but not on ARTs, CD4 levels decline thereby causing the immune system to weaken and resulting in progression to AIDS and death from AIDS related diseases [5]. The benefits of diagnosing HIV early include accessing HIV treatment and care promptly. Accessibility of ART to all people living with HIV is still a challenge in low- to mid-income countries and this can be addressed by improving the access to HIV testing and ensuring that those that require treatment are placed on treatment. South Africa has the highest percentage of HIV infected people, however only 29% of people in low income townships are virologically suppressed showing that more work is required in diagnosing, treating and monitoring HIV infected people in these communities [6].

There is a growing demand to improve and simplify the effectiveness of HIV diagnostics without compromising the quality of patient care. To increase the accessibility of treatment for HIV infected patients residing in resource limited settings of developing countries, a lot of work has been directed to developing point of care (POC) that meet the “ASSURED” characteristics set out by the World Health Organization (WHO), which outline standards for evaluating POC diagnostics. These are diagnostics that are **A**ffordable, **S**ensitive, **S**pecific, **U**ser-friendly, **R**apid and robust, **E**quipment-free and can be **D**eliverable to the users [7]. The majority of testing platforms are centralised, laboratory based and require trained laboratory staff unlike POC instruments that do not require trained laboratory personnel and facilities to obtain a diagnostic result. In the case of HIV, the analytical targets can include human cells, proteins as well as nucleic acids and the samples can be blood, saliva and urine [8]. Irrespective of where these POC tests are used, they allow a sample with little or no preparation and the results can be obtained in seconds to a few hours [8].

Currently, CD4 testing is being used as a determining factor for eligibility of individuals infected with HIV to treatment and care. The treatment eligibility threshold levels were set at 200 cells/mm^3^ from the year 2002 which was later changed to 350 cells/mm^3^ from 2010 onwards [9]. The antiretroviral guidelines from the WHO for 2013 recommends initiating ART in HIV infected adults having a CD4 count of 500 cells/mm^3^ or below [10]. The test and treat strategy is currently being evaluated and debated in many countries with some already practising it. In South Africa, a country with the world’s largest programme for ART, the “test and treat” strategy was implemented from September 2016 onwards. Unlike CD4 count, HIV viral load is used when assessing response of HIV infected people to ART and success to the treatment is indicated by an undetectable viral load result. Many studies have shown that people with HIV that are receiving ART can have an undetectable viral load, however the CD4 count does not decrease over time [11]. A study by Rutherford et al. showed that in cases where viral load is performed routinely, the rate of CD4 testing can be reduced or completely ceased [12].

Researchers globally are working on platforms and tests to address the gap of POC devices that fully satisfy the ASSURED characteristic and can be used in resource limited settings. In this review, we begin our discussion with the different stages involved in HIV infection as different diagnostic tests are used in these stages. We then go into the HIV diagnostics where we provide detail on HIV rapid tests, viral load monitoring and CD4 counting with specific reference to POC technologies. Lastly, we provide the technical challenges involved in the development and commercialisation of POC devices and then give the future perspective.

## 2. Stages of HIV Infection

**Figure 1:**
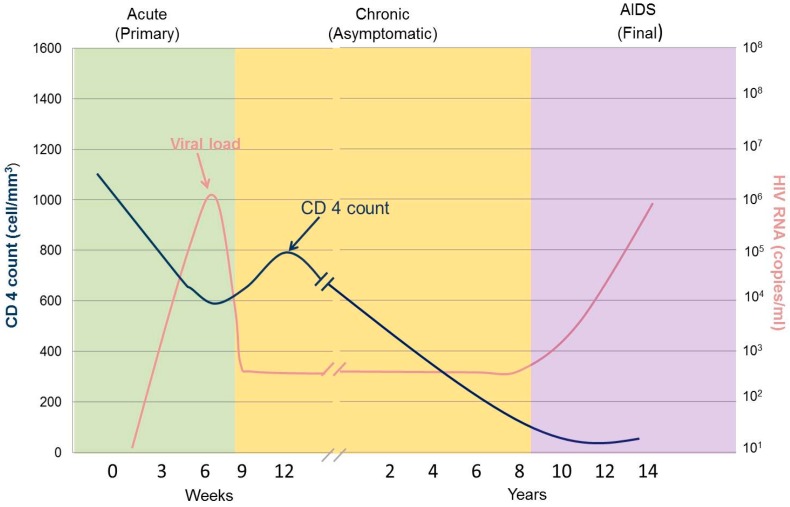
An illustration of the different stages of HIV infection.

Since HIV infection has three distinct stages, the acute (primary), chronic (asymptomatic) and AIDS (final) stages, different techniques/test types are utilised to detect the presence of an infection at each stage [13]. Acute phase is the earliest phase which generally develops within two weeks after a person is infected with HIV and at this stage many infected individuals experience flu-like symptoms, with reduction of CD4 positive cells as the virus multiplies rapidly (Figure 1) [13,14]. In the acute phase, infected individuals are highly infectious and the virus transmission rate is high [15]. When the immune system starts to recover, it fights to reduce viral levels, but CD4 counts do not usually recover to levels similar to pre-infection levels. The HIV antibodies are detectable after two weeks post infection, however prior to the presence of antibodies, infection is detected by p24 test and nucleic acid tests [16].

The next phase is the chronic (asymptomatic phase) in which HIV continues to multiply and CD4 cells are reduced at slower rate compared to the acute phase; the virus is still transmittable. Without anti-retroviral therapy, the infection progresses to AIDS in 10 years, however progressions can occur faster in some individuals. During this phase the diagnosis is mainly done using antibody detecting immunoassays [17]. The final phase is AIDS, which is highly symptomatic as the CD4 is reduced to less than 200 cells/mm^3^ [18]. The immune system of a healthy individual has CD4 counts between 500 and 1600 cells/ mm^3^ (aidsinfonet.org). In the AIDS stage, infected individuals are prone to opportunistic infections and in the absence of therapy, death occurs [5].

## 3. HIV Diagnostic Tests

HIV diagnostic tests can either detect virus molecules (HIV RNA and p24 antigen) or antibodies to the virus [19]. Detecting the p24 antigen and antibodies involves immunoassays, whereas the HIV RNA is detected using nucleic acid tests [20]. The standard method of testing for HIV such as Enzyme Linked Immunosorbent Assay (ELISA) as well as the western blot technique and confirmation test (p24 antigen or viral nucleic acid test) usually takes numerous days before a diagnostic result is ready [21]. Along with time delays, tests such as the nucleic acid tests are expensive, complex and require skilled laboratory staff and laboratory settings and therefore are not suitable for resource limited settings [22]. Such delays result may result in the failure of following up of patients in resource limited settings therefore POC testing can be useful in addressing the delay in detection by providing preliminary antibody results.

### 3.1. HIV Rapid Tests

A variety of rapid tests for diagnosing HIV in resource limited settings are commercially available. Nonetheless, not all rapid tests can be applied at the POC (e.g., some may require serum separation prior to using) [21]. All the approved HIV POC tests should have a sensitivity and specificity matching the ELISA kits that are used in laboratories. The most frequently used rapid tests are the lateral flows that require few or no reagents and are generally cheap and results are obtained within 30 min [23]. Rapid tests can detect antibodies to HIV from a minute volume of whole blood, serum, plasma, urine or saliva. For example, the OraQuick Rapid HIV-1/2 antibody test is a POC test that can detect both HIV-1 and HIV-2 in whole blood or plasma and oral fluid specimens at low concentrations. The OraQuick In-home HIV test is the first FDA approved over the counter rapid test for detection of HIV antibodies [21]. Rapid tests for HIV have evolved to detect a combination of tests such as the p24 antigen and the HIV antibody, as well as tests for early infant diagnosis. Fourth generation HIV rapid test can detect both the antigen and antibodies (e.g., ARCHITECT HIV Ag/Ab combo and Alere Determine HIV 1/2 Ag/Ab combo). The latter was the first rapid POC test capable of detecting both the HIV 1/2 antibodies and the antigen. 

### 3.2. HIV Viral Load Measurement

HIV viral load is the most effective method to evaluate the response of ART on HIV infected patients and is seen as the gold standard to monitor clinical prognosis of patients on ART. The threshold used to detect the failure to ART was reduced from 5000 to 1000 virus copies/mL in South Africa in April 2010, and in June 2013 the WHO updated their guidelines to match that of South Africa [24]. HIV viral load assays can be classified as nucleic acid based tests or non-nucleic acid based tests. The nucleic acid based tests detect and measure viral RNA, while the non-nucleic acid tests detect and quantify viral enzymes and proteins that have a direct correlation with amount of viral RNA. The majority of commercially available instruments are nucleic acid tests and examples include: Versant HIV-1 RNA 3.0 Assay, NucliSens HIV-1 Assay, LCx HIV RNA quantitative assay, RealTime HIV-1 (m2000rt), and Cobas Taqman HIV-1 test. These tests utilize varying amplification principles such as branched DNA (bDNA), polymerase chain reaction (PCR) and nucleic acid sequence based amplification to quantify the viral RNA [25]. An example of non-nucleic acid based assay is the ExaVir load assay which measures the HIV reverse transcriptase activity in plasma to determine the HIV viral load [4]. The above mentioned nucleic acid based and non-nucleic acid based tests are centralised and require skilled laboratory technician to operate the instruments. Several POC devices for viral load are currently available and in the pipeline, and will be discussed below (Table 1).

#### 3.2.1. Simple Amplification Based Assay (SAMBA)

The SAMBA (Diagnostics for the real world) was developed at the Diagnostics Development Unit (DDU) in the University of Cambridge, by a team lead by Dr Helen Lee. SAMBA is an innovative nucleic acid based rapid test suitable for acute HIV infection, early infant diagnosis and viral load monitoring. This bench-top analyser integrates the extraction, amplification and detection with the detection of the amplified products occurring in a closed cartridge [2]. The instrument utilizes 100 μL whole blood or 200 μL of plasma and the preparation of sample involves cell lysis and nucleic acid extraction. A target sequence is captured by a capture probe and a detector probe labelled with multiple hapten moieties is subsequently bound to the target sequence allowing amplification of the signal. The detection of the DNA or RNA can be read off visually within 25 min in a dipstick format on the nitrocellulose membrane [4]. The instrument can differentiate between HIV infected patients having a viral load of below or above 1000 copies/mL. An evaluation of SAMBA in Malawi, London and Uganda showed that SAMBA has sufficient correlation with the standard measurement techniques for HIV viral load [26].

#### 3.2.2. EOSCAPE HIV

The EOSCAPE-HIV (Wave 80 Biosciences, San Francisco, CA, USA) is a nucleic acid based technology designed for usage in resource limited settings to detect HIV-1 RNA. This assay uses 100 μL of whole blood or plasma to quantitate HIV RNA within 50 min [27]. Each phase towards a result, including DNA/RNA extraction, takes place inside the EOSCAPE cartridge containing all the necessary reagents to perform the test. The unit is battery powered which can last for up to 8 h and has a small portable touchscreen for displaying the results [28]. The EOSCAPE is capable of either providing quantitative or qualitative HIV-RNA with a limit of detection of a 1000 copies/mL.

#### 3.2.3. Liat™ Analyser

The Liat™ Analyser (IQuum, Inc, Marlborough, MA, USA) is an easy to use, fully automated, rapid and sensitive test for quantifying HIV RNA in plasma or whole blood. This analyser integrates sample processing and detection, including extraction of nucleic acid, purification, reverse transcription and real time PCR takes place in the in a closed tube format [29]. When performing the test, the Liat tube which is inserted into the analyser has sample metering capabilities to confirm that the sample is the accurate volume [28]. The Liat tube also contains an internal control which is analysed with the sample to ensure that all the necessary steps are performed. All the reagents are pre packed inside the Liat tube segments in their desired volumes partitioned by peelable seals following a logical sequence [29]. The Liat analyser allows automation of the nucleic acid test from sample collection to result in less than an hour. A laboratory evaluation of the Liat analyser showed that the instrument can be interchanged with available viral load technologies in South Africa [30].

#### 3.2.4. Alere™ q Analyser

The Alere™ q analyser (Alere Inc, Waltham, MA, USA) is an automated cartridge based nucleic acid testing platform which enables POC testing and could be used for early infant diagnosis. This system uses 25 μL of whole blood or plasma and has the ability to distinguish between HIV-1 groups O and M/N and HIV-2 [31]. The sample is loaded into the cartridge and the cartridge cap is snapped into place to avoid any chance of spillage and contamination. All the reagents are contained within the cartridge which is single use. The instrument then automatically performs the isolation of RNA, reverse transcription and amplification, and finally the detection of PCR products [31]. This battery powered instrument was designed for using in resource limited settings. Jani et al. showed that Alere™ q can adequately predict ART failure, having a threshold of 1000 copies/mL with a sensitivity of 84% and specificity of 90.3% [32]. An evaluation study comparing Alere™ q analyser with a traditionally used Roche CAP/CTM HIV PCR for early infant diagnosis in South Africa indicated that Alere™ q analyser performs well for early infant diagnosis and showed high specificity [33].

#### 3.2.5. GeneXpert^®^ System

The GeneXpert^®^ System (Cepheid, Sunnyvale, CA, USA) is an automated qualitative test used for detecting HIV-1 total RNA in blood spots and whole blood. This system integrates and automates preparation of the sample, extraction of nucleic acid and amplification, and the detection of the sequence of interest with real time reverse transcription PCR (RT-PCR) [34]. It uses a single use disposable cartridge containing all the reagents necessary for the RT-PCR procedure. This cartridge minimises cross contamination between samples as it is self-contained. An internal control is included in the reagents to ensure adequate processing of the target and to monitor the PCR process. An evaluation of the GeneXpert for HIV-1 in Israel showed a sensitivity and specificity of 100% in samples of patients with a known HIV-1 status [35].

### 3.3. Early Infant Diagnosis of HIV

Diagnosing HIV infection in infants early can be of great benefit in reducing the mortality of infants due to the HIV infection. The maternal antibodies presence in the system of infants makes the antibody test undesirable for diagnosing HIV in infants less than the age of 18 months. This therefore means that technologies that detect viral components are required for early infant diagnosis and this can include assays that detect: viral capsid p24 antigen, cell free RNA or viral DNA incorporated into the host [36,37]. Nucleic acid amplification based tests use viral RNA as a template, which gets converted to cDNA and they are more sensitive than p24 based assays [38]. There are commercially available lab based tests used for qualitative HIV-1 viral DNA, e.g., HIV Proviral DNA PCR Kit (Norgen Biotek Corp, Thorold, ON, Canada), HIV-1 DNA, Qualitative, PCR (Quest Diagnostics, Secaucus, NJ, USA), Real Time HIV-1 Qualitative HIV-1 PCR (Arluplab, Salt Lake City, UT, USA), and Qualitative (Abbott, Chicago, IL, USA). The current p24 immunoassays show more sensitivity than the previously used p24 tests because of the incorporation of a denaturing step that separates the p24 antibody complexes which interferes with the sensitivity of the assay [4]. However, similar to nucleic acid amplification based assays, p24 immunoassays require specialized equipment and skilled technicians to perform the test. In order to perform these tests in resource limited settings, early infant diagnosis rapid tests are required.

### 3.4. CD4 Tests

Similar to HIV viral load, CD4 count monitors disease progression in individuals infected with HIV as it is a critical indicator of immune function. In instances where “test and treat” is used, CD4 count will still be critical in the baseline evaluation of patients to inform initial clinical management decisions, especially those presenting late for treatment and those that are on ART where virological failure or clinical deterioration is present or suspected [39]. CD4 count still remains the best option for determining the patient’s immunological status, prioritization of patients for testing of opportunistic infections such as *Cryptococcus* and for prioritizing patients for ART in places where test and treat is not yet used. Although HIV viral load is an excellent tool for determining viral suppression, it cannot replace CD4 count; however, the two techniques are complementary.

HIV targets the key cells of the immune system, the CD4+ T cells which are required to fight infection [40]. Their depletion results in immune system failure and vulnerability to any form of infection. The levels of CD4+ cells can be expressed as an absolute count for specified volume, as a percentage of the total lymphocyte population or as a ratio with another subset of lymphocyte [41]. CD4 absolute counts are suitable for evaluating the status in adults while for children it is ideal to measure the percentage of the CD4 in the total population of lymphocytes or the ratio of CD4/CD8 lymphocytes because lymphocytes population such as CD4+ are greater in children [41]. Most methods that are used to assess CD4+ cells make use antibodies specific to human CD4 for cell labelling, strategies to capture cells or both for subsequent detection. Fluorescence activated cell sorting (FACS) using flow cytometry has been the golden standard for CD4 measurement. Flow cytometry instruments can either be a single platform or a dual platform. Single platform based instruments can measure the absolute CD4 in a precise volume of sample or measure the absolute number of CD4 cells from a ratio of known concentration of beads to CD4+ cells [42]. Examples of single platform laboratory based instruments include; FACSCalibur (Becton Dickinson, NJ, USA), FACSCount, Partec CyFlow Counter (Partec, Münster, Germany) and Apogee Auto40 (Apogee systems, Cape Canaveral, FL, USA). In dual platforms, a flow cytometer is combined with a haematological analyser to measure the absolute CD4 count that is the fraction of CD4 in a small percentage of leukocytes derived from FACS multiplied by the total leukocyte population established with the haematological analyser [40]. Below, we discuss in detail the CD4 POC instruments that are commercially available and some which are still in the pipeline. These CD4 POC devices are shown in Table 2.

#### 3.4.1. PointCare NOW™

PointCare NOW™ (PointCare Technologies Inc, Marlborough, MA, USA) is a portable flow cytometry based diagnostic instrument which is specifically designed for HIV/AIDS POC use in decentralized settings [43]. This instrument is the only POC instrument with FDA clearance and generates a test result in 8 min [41]. Single cells flow through the focal point of a light emitting diode (LED) and the instrument detects light scatter and light blockage by the cells. The instrument uses the principle found in modern haematological analysers to distinguish and counts all the classes of white blood cells using the light scatter [43]. CD4 lymphocyte counting using the light scatter is accomplished with a CD4 monoclonal antibody labelled with colloidal gold which binds to the surface of CD4 lymphocytes. The CD4 lymphocytes bound to the antibody scatter generate more light because they are covered with gold nanoparticles and can be easily identified by the systems and counted. The evaluation of PointCare NOW™ in South Africa, Mozambique and Canada showed that the instrument performed inadequately in absolute and percentage CD4 enumeration and was not effective in the clinical management of HIV in adults [44]. A similar study in children using a small sample size was not adequate to draw a conclusion. The major drawback of this device is that it is not compatible with commonly used external quality controls, as quality evaluation is of critical importance in implementing decentralized testing [45].

#### 3.4.2. CyFlow^®^ miniPOC

CyFlow^®^ miniPOC (Partec GmbH, Münster, Germany) is also a portable flow cytometry based diagnostic instrument for monitoring HIV/AIDS in remote areas and health care centres. This instrument can analyse a maximum of 250 CD4/CD4% tests daily and the detection range of CD4 is from 0 to 5000 μL^−1^ [28]. Since this instrument can detect both absolute CD4 and CD4 percentage it can be used in both adults and infants. The CD4+ detection reagents are stored dry thereby eliminating the need for cold storage. To perform the test, 20 μL of whole blood is introduced into the tube filled with Partec reagent and incubated for 15 min before the buffer is added and loading sampling device into the instrument. An evaluation of the instrument on HIV infected patients attending university hospital in Dakar showed that the CyFlow miniPOC CD4 results correlated with that of FACSCount and FACSCalibur CD4 [46]. The CyFlow^®^ miniPOC showed reliable results in both the CD4 percentages and absolute CD4 counts even when it was used in field conditions and could be used in resource limited settings for monitoring patients with HIV. The limitations of this instrument are that it requires expensive equipment, instrument maintenance and adequate operation training thereby limiting the widespread of this instrument in resource limited settings [47].

#### 3.4.3. Daktari™ CD4 Counter

Daktari™ CD4 counter (Daktari Diagnosis, Inc, Cambridge, MA, USA) is label free and functions without optics, lenses or cameras, hence it is very portable and robust. Daktari utilizes affinity chromatography where CD4+ cells are specifically captured from whole blood in a controlled shear stress microfluidic system [48]. The test has a reader and a disposable blister cassette which contains all the reagents in blister packs. As the blood flows through the system, the CD4+ cells are captured by the surface immobilized anti-CD4 antibodies while large sized monocytes fail to be captured because of the large shear forces. Cell lysis and the release of cellular components occur after two reagents have been released into the cell binding chamber and this is then measured by impedance spectroscopy [42].

#### 3.4.4. MBio™Diagnostics CD4 System

This imaging cytometry technology utilizes disposable single use cartridges and a simple reader with immunostaining of whole blood taking place in the cartridge [41]. Capillary or venepuncture whole blood is delivered into the cartridge containing all the necessary reagents. The lyophilized reagents incorporated in the cartridge eliminate the need for cold storage and facilitates a single step test process. The lyophilized reagents are stored dry thereby eliminating the need for cold storage. The lyophilized reagents have two fluorescently labelled antibodies to perform a two-colour analysis of the image of the stained whole blood resulting in the absolute count of CD3+ and CD4+ cells [42]. MBio system (Boulder, CO, USA) has a multirack-cartridge rack that allows parallel processing; this is possible because sample processing of the blood takes place the disposable cartridge and not in the reader. The system has a turnaround time of approximately 20 min per sample and the throughput capacity is 15 samples per hour and 100 samples per day [28]. A validation study conducted by Logan et al. using a conventional flow cytometer and the MBio system showed close correlation in the results from the same participants when tested with the two systems in different locations using different operators [48]. The results obtained suggested that the MBio system possesses great potential as a POC device for quantification of CD4 cells in resource limited settings [49].

#### 3.4.5. Visitect CD4

Visitect CD4 (Omega Diagnostics, Alva, UK), a rapid disposable semi quantitative CD4 test, was launched in Washington D.C. at the 2012 AIDS conference and was developed by the Burnet Institute in collaboration with Omega Diagnostics Group. This test measures the CD4 protein on the T cells and not the CD4 cells directly. Visitect CD4, determines CD4+ T cells in 40 μL of whole blood and is based on a lateral flow technology. The red blood cells and monocytes from the whole blood are held on a sample pad where the sample was applied while other cells travel through the chip. CD4+ cells are immobilised by binding them to biotin labelled anti-CD4 antibodies, which is detected by a colloidal gold labelled anti-biotin [41]. The result of the semi-quantitative test is easy to interpret and provides a visual readout “treat or no treat” in less than 40 min. Treat is when the “T” line is equal or weaker than 350 line (CD4+ T cell count is ≤350 μL) while no treat is when the “T” line is stronger than 350 line (CD4+ T cell count is >350 μL).

#### 3.4.6. BD FACSPresto

The BD FACSPresto (BD Biosciences, NJ, USA) is a near patient CD4 counter instrument which provides absolute CD4, percentage CD4 and Haemoglobin (Hb) concentration. Whole blood is introduced into the cartridge, which contains dried fluorochrome conjugated antibody reagents [50]. The blood reacts with the reagents and the antibodies then binds to the surface antigens on the T lymphocytes and monocytes. The cartridge is incubated for 18 min outside the counter for staining to occur and this can increase the throughput and an operator can run up to 60 tests in an 8 h shift. Post incubation, the stained cartridge is inserted into the instrument and the integrated software identifies the population of interest and counts the absolute and percentage CD4+ T lymphocytes and calculates the Hb concentration. A comparison study between several existing technologies and the BD FACSPresto using 264 HIV positive patients in two rural health care facilities showed that the FACSPresto functioned better in the laboratory setting than the conventional standard technologies, which can result in up to 20% of patients requiring treatment being missed [51].

#### 3.4.7. Zyomyx CD4 Test

This instrument free POC was developed by Zyomyx Inc. and was funded by the Bill and Melinda Gates foundation [52]. The test consists of a single disposable cartridge and a mechanical mixer/spinner. Whole blood is placed into the cartridge, where the CD4 cells from the blood sample binds to the heavy anti-CD4 antibody coated particles [2]. To enable a visual readout at the end of the protocol, anti-CD4 antibody coated particles are optically dense. The mechanical mixer spins the cartridge enabling only conjugated cell pass through and enter the capillary tube which has high density medium resulting in the formation of a cell stackwidth. The height of the stacked beads which are bound to the cells is directly proportional to the number of CD4 cells and can be read with assistance of a magnifying lens. The capillary tube has a calibrated scale that shows the cell concentration similar to reading a thermometer. The system also contains anti-CD14 which binds to monocytes and this is included to reduce contamination by monocytes.

#### 3.4.8. Pima™ Analyser

The Pima™ Analyser (Alere Inc, Waltham, MA, USA) for CD4 testing is the first commercially available instrument that does not use the traditional flow cytometry principle and was released in 2009. Pima CD4 is an image based immune haematology test that measure absolute count of CD3+/CD4+ cells and comprises of the analyser and a disposable Pima test cartridge which contains dried reagents needed to perform the test [53]. Approximately 25 μL of whole blood is inserted into the cartridge and the blood moves by peristalsis into the incubation platform where the blood interacts with two antibodies (anti-CD3 and anti-CD4) attached to a flourochrome. After the incubation period, the cartridge is then loaded into the instrument, where excitation of the fluorophores by the light emitting diodes takes place [42]. An on-board CCD camera picks up the fluorescence signals, and the image analysis software detects and counts the T-helper lymphocytes that express both antigens (CD3/CD4). T-helper lymphocytes express both CD3 and CD4 surface antigen while other blood cell types can express either CD3 or CD4. A Performance Evaluation of the Pima™ Analyser in 203 HIV infected patients infected individuals in resource limited settings showed good reproducibility of CD4 cell count results which were in agreement with the traditional dual platform and bead based flow cytometers [54]. However, the need for basic laboratory infrastructure in order to run this instrument impedes it from being in resource limited settings. It was also noted that this instrument is affected by variation in the capillary sampling.

## 4. Technical and Commercialization Challenges in POC Diagnostics for HIV

Despite the recent advances in the field of engineering, material science and nanotechnology in developing POC diagnostics, there are no instruments that meet the ASSURED criteria. The integration of microfluidic systems into POC technologies also remains a challenge due to difficulty in the miniaturization of various components required for active microfluidic transport. In order for POC instruments to be useful, they generally need to perform the following procedures: (1) sample collection; (2) sample processing; (3) testing; (4) data analysis and interpretation of the data; and (5) waste disposal [47]. The testing procedure exhibit great challenges in the use of POC diagnostics in resource limited settings especially in the rural areas because of lack of trained personnel and limited laboratory testing capability. Most resource limited settings lack electricity, cold chain abilities and the temperature is usually higher than the room temperature. To overcome these limitations, reagents should be stable at high temperatures, making the dry form of reagents more favourable and analysers should operate using a battery or solar power [55].

Another challenging factor is the sample collection and preparation, although some companies such as the Alere, Becton Dickinson and Cepheid have made significant progress in the enabling of effective sample collection. Daktari™ CD4 counter has also eliminated the sample preparation step but as a result of the shifting global landscape around CD4 counting, the company has discontinued the counter cartridges. Another important aspect of POC instruments that is often neglected by POC developers is the user-interface. Results generated by POC instruments should be simple to understand and easily interpretable by less skilled health workers and even patients at home [47]. With respect to transport, mechanical vibrations during transportation may damage sensitive parts of the POC device and should also be taken into consideration during development of the device. Furthermore, user needs, which are critical in the product development decision making, should be included in R&D and trial phases.

Prior to the translation of POC technologies into commercially viable products, the technologies need to demonstrate high analytical precision and reproducibility for analyte quantification in a large number of patient’s samples without significant interference from non-specific substances. Furthermore, the instruments need to be validated by using accredited instruments. Another factor which determines the commercialization and wide-scale adoption of POC technologies is the economic feasibility [56]. Generally, the commercialisation process of new diagnostic devices is lengthy and very expensive. On average, it is estimated that the product development process can take up to 10 years and a cost of $100 million (USD) from proof of concept to product launch [57]. Even though many companies have managed to enter their POC technologies into the market, a number of promising technologies have failed at market entry. We envisage that the current technical and commercialization challenges will persist due to the barriers that have been mentioned. However, in the in the long run, many POC devices will enter the market.

## 5. Future Prospects

There is a strong need for the development of HIV diagnostics that rapid, inexpensive and simple for application in resource limited settings. POC diagnostics have been undoubtedly a dream companion for medical professionals in resource constrained settings because of their low cost; they do not require skilled lab technicians; and they can generate a test result rapidly. The aforementioned mentioned POC technologies excel in certain aspects but fail to meet every aspect of the ASSURED characteristic set out by the WHO. This therefore implies that there is a strong need to further improve the currently available systems. Most of these devices are benchtop sized with the cost of the analyser ranging approximately $3000–$10,000 [58]. Integration of smartphones with microfluids and paper based platforms hold great promise in the quest of developing POC devices. Wearable or implantable sensors also holds great promise in the development of POC diagnostics for HIV diagnostics and monitoring individuals on ART in resource limited settings. Researchers are collaborating towards the development of POC device and companies are either developing new devices or investing in improving current technology.

## 6. Conclusions

The advancement in technology will eventually result in the development of POC technologies that meet the ASSURED criteria. The innovative platforms or assays mentioned in this review use simple and inexpensive components, and are cassette based, battery powered and portable. Furthermore, they can easily be used and in general they do not need skilled personnel and reagents are temperature insensitive. The progress made in the development of these POC devices for HIV expands access to testing in remote and rural settings, leading to rapid diagnosis and eventually ART treatment.

## Figures and Tables

**Table 1:** Point of care technologies for viral load measurement.

Viral Load Tool	POC	Analyte	Detection Method	Time to Result	Sample Type and Volume	Additional Information
SAMBA	Yes	RNA	Isothermic amplification and Hapten-based signal detection	90 min (SI 4 sample throughput, 24–48 tests/day), (SII 4 tests a day)	Plasma: 200 μL	Semi-quantitative; Detection: 1000 copies/mL Regulatory status: CE marked
EOSCAPE HIV	Yes	RNA	Amplification/Detection	50 min	Whole blood: 100, plasma: 100 μL	Qualitative and quantitative Detection threshold: 1000 copies/mL Regulatory status: NA
Liat analyser	Yes	RNA	Multiplex Real-Time PCR Amplification/Detection	30–35 min	Whole blood: 75 μL, plasma: 150 μL	Quantitative Detection threshold: 1000 copies/mL Regulatory status: In development
Alere q analyser	Yes	RNA	Real time PCR/microprobe array	52 min (8 h throughput, 8 tests)	Whole blood: 25 μL; plasma: 25 μL or 500 μL	Quantitative Detection threshold: 1000 copies/mL Regulatory status: CE marked
GeneXpert	Near POC	RNA	Multiplex Real-Time PCR NA Amplification/Detection	90 min	Plasma: 1 mL	Quantitative Detection range: 40–10,000,000 copies/mL Regulatory status: CE marked

CE: Conformite Europeene; NA: not available.

**Table 2:** Point of care CD4 technologies.

CD4 Tool	Analyte	Detection Method	Technology	Time to Result or Tests	Additional Information
PointCare NOW™	CD4, CD4%, 12 blood parameters, haemoglobin	CD4 monoclonal antibody labelled with colloidal gold	Flow cytometry	8 min	Measures blood, fully automated, portable, table top Regulatory status: FDA approved CE marked
CyFlow^®^ miniPOC	CD4, T cell, CD4%	Antibodies	Flow cytometry including laser modules, Optics, fluidics and electronics	250 tests per day	Blood samples can be run one at a time or in batches, portable, compact Regulatory status: CE marked
Daktari™ CD4 Counter	CD4	Antibody, label free, functions without optics, lenses or cameras	Microfluidic cell chromatography and lysate impedance spectroscopy	14 min	Portable, robust Regulatory status: NA
MBio™Diagnostics CD4 System	CD4 and CD3	Labelled antibodies	Imaging cytometry, immunostaining	+/−20 min per sample	Robust, uses low cost lasers Regulatory status: NA
Visitect CD4	CD4 on T cells	Antibodies, colloidal gold	Lateral flow	40 min	Semi-quantitative CD4 > 350, CD4 > 500 Regulatory status: CE marked
BD FACSPresto	CD4, %CD4 and haemoglobin	Fluorochrome conjugated antibody	Image based counting technology	60 tests in 8 h	Cartridge contains dried reagents Regulatory status: CE marked WHO prequalification
Zyomyx CD4 Test	CD4	Antibodies	Mechanical mixer/spinner, capillary tube	6 min	Instrument free Regulatory status: CE marked
Pima™ Analyser	CD4, CD3	Antibodies	Image based immune haematology test, fluorescence	20 min	Good technical support, no routine maintenance Regulatory status: WHO prequalification

FDA, Food and Drug Administration; CE, Conformite Europeene; NA, not available; WHO, World Health Organisation.

## References

[1] Barre-Sinoussi F., Cherman J.C., Rey F., Nugeyre M.T., Chamaret S., Gruest J., Dauguet C., Axler-Blin C., Vézinet-Brun F., Rouzioux C. (1983). Isolation of a T-lymphotropic retrovirus from a patient at risk for acquired immune deficiency syndrome (AIDS). Science.

[2] UNAIDS: Fact Sheet 2015 (2015). UNITAID. HIV/AIDS Diagnostics Technology Landscape; Semi-Annual Update. http://www.unaids.org/sites/default/files/media_asset/20150901_FactSheet_2015_en.pdf.

[3] Stevens W., Gous N., Ford N., Scot L.E. (2014). Feasibility of HIV point of care tests for resource limited settings: Challenges and solutions. MBC Med..

[4] Setty M.K.H.G., Hewlett I.K. (2014). Point of care technologies for HIV. AIDS Res. Treat..

[5] World Health Organization (2005). Interim WHO Clinical Staging of HIV/AIDS and HIV/AIDS Case Definitions for Surveillance, Africa Region. http://www.who.int/hiv/pub/guidelines/clinicalstaging.pdf?ua=1.

[6] Kranzer K., Lawn S.D., Johnson L.F., Bekker L., Wood R. (2013). Community viral load and CD4 count distribution among people living with HIV in a South African township: Implications for treatment as prevention. J. Acquir. Immune Defic. Syndr..

[7] Peeling R.W., Holmes K.K., Mabey D., Ronald A. (2006). Rapid tests for sexually transmitted infections (STIs): The way forward. Sex. Transm. Infect..

[8] Gubula V., Harris L.F., Ricco A.J., Tan M.X., Williams D.E. (2012). Point of care diagnostics: Status and future. Anal. Chem..

[9] (2010). World Health Organization: Antiretroviral Therapy for Infection in Adults and Adolescents: Recommendations for a Public Health Approach. http://apps.who.int/iris/bitstream/10665/44379/1/9789241599764_eng.pdf.

[10] (2015). World Health Organization: Guideline on When to Start Antiretroviral Therapy and on Pre-Exposure Prophylaxis for HIV. http://apps.who.int/iris/bitstream/10665/186275/1/9789241509565_eng.pdf.

[11] Gale H.B., Gitterman S.R., Hoffman H.J., Gordin F.M., Benator D.A., Labriola A.M., Kan V.L. (2013). Is frequent CD4+ T-lymphocyte count monitoring necessary for persons with ≥300 cells/μL and HIV-1 suppression?. Clin. Infect. Dis..

[12] Rutherford G.W., Angelmyer A., Eastbrook P.J., Horvath T., Vitoria N., Penazzato M., Doherty M.C. (2014). Predicting treatment failure in adults and children on antiretroviral therapy: A systemic review of the performance characteristics of the 2010 WHO immunologic and clinical criteria for virologic failure. AIDS.

[13] Enger C., Graham N., Peng Y., Chmiel J.S., Kingsley L.A., Detels R., Muñoz A. (1996). Survival from early, intermediate, and late stages of HIV infection. JAMA.

[14] Celum C.L., Buchbinder S.P., Donnell D., Douglas J.M., Mayer K., Koblin B., Marmor M., Bozeman S., Grant R.M., Sheppard J.F.H.W. (2001). Early human immunodeficiency virus (HIV) infection in the HIV Network for Prevention trials vaccine preparedness cohort: Risk behaviours, symptoms, and early plasma and genital tract virus load. J. Infect. Dis..

[15] Jacquez J.A., Simon C.P., Koopman J.S., Sattenspiel L., Perry T. (1988). Modeling and analyzing HIV transmission: The effect of contact patterns. Math. Biosci..

[16] Schacker T., Collier A.C., Hughes J., Shea T., Corey L. (1996). Clinical and epidemiologic features of primary HIV infection. Ann. Intern. Med..

[17] Roederer M., Dubs J.G., Anderson M.T., Raju P.A., Herzenberg L.A., Herzenberg L.A. (1995). CD8 naive T cell counts decrease progressively in HIV-infected adults. J. Clin. Investig..

[18] Margolick J.B., Donnenberg A.D., Chu C., O’Gorman M.R., Giorgi J.V., Munoz A. (1998). Decline in total T cell count is associated with onset of AIDS, independent of CD4(+) lymphocyte count: Implications for AIDS pathogenesis. Clin. Immunol. Immunopathol..

[19] Gurtler L. (1996). Difficulties and strategies of HIV diagnosis. Lancet.

[20] Zhang M., Versalovic J. (2002). HIV update: Diagnostic tests and markers of disease progression and response to therapy. Am. J. Clin. Pathol..

[21] Arora D.R., Maheshwari M., Arora B. (2013). Rapid point of care testing for detection of HIV and clinical monitoring. ISRN AIDS.

[22] Aleku G.A., Adoga M.P., Agwale S.M. (2014). HIV point of care diagnostics: Meeting the special needs of sub-Saharan Africa. J. Infect. Dev. Ctries..

[23] Wu G., Zaman M.H. (2012). Low-cost tools for diagnosing and monitoring HIV infection in low-resource settings. Bull. WHO.

[24] WHO (2013). Consolidated Guidelines on the Use of Antiretroviral Drugs for Treating and Preventing HIV Infection. http://www.who.int/hiv/pub/guidelines/arv2013/en/.

[25] Luft L.M., Gill M.J., Church D.L. (2011). HIV-1 viral diversity and its implications for viral load testing: Review of current platforms. Int. J. Infect. Dis..

[26] Ritchie A.V., Ushiro-Lumb I., Edemaga D., Joshi H.A., De Ruiter A., Szumilin E., Jendrulek I., McGuire M., Goel N., Sharma P.I. (2014). SAMBA HIV semiquantitative test, a new point of care viral load monitoring assay for resource limited setting. J. Clin. Microbiol..

[27] Innovations in Molecular Diagnostisc. http://www.wave80.com/products/.

[28] Murtagh M. (2012). UNITAIDS Technical Report: HIV/AIDS Diagnostic Landscape.

[29] Tanriverdi S., Chen L., Chen S. (2010). A rapid and automated sample to result HIV load test for near patient application. J. Infect. Dis..

[30] Scott L., Gous N., Carmona S., Stevens W. (2015). Laboratory Evaluation of the Liat HIV Quant (IQuum) Whole-Blood and Plasma HIV-1 Viral Load Assays for Point-of-Care Testing in South Africa. J. Clin. Microbiol..

[31] (2016). World Health Organization: WHO Prequalification of In Vitro Diagnostics, PUBLIC REPORT. http://www.who.int/diagnostics_laboratory/evaluations/pq-list/hiv-vrl/160613PQPublicReport_0226-032-00AlereHIVDetect_v2.pdf?ua=1.

[32] Jani I.V., Meggi B., Vubil A., Sitoe N.E., Bhatt N., Tobaiwa O., Quevedo J.I., Loquiha O., Lehe J.D., Vojnov L. (2016). Evaluation of the Whole Blood Alere Q NAT Point-of-Care RNA Assay for HIV-1 Viral Load Monitoring in a Primary Health Care Setting in Mozambique. J. Clin. Microbiol..

[33] Hsiao N., Dunning L., Kroon M., Myer L. (2016). Laboratory Evaluation of the Alere q Point-of-Care System for Early Infant HIV Diagnosis. PLoS ONE.

[34] (2016). World Health Organization: WHO Prequalification of In Vitro Diagnostics, PUBLIC REPORT. http://www.who.int/diagnostics_laboratory/evaluations/pq-list/hiv-vrl/160613PQPublicReport_0259-0700-00_XpertQualHIV_v2.pdf?ua=1.

[35] Michaeli M., Wax M., Gozlan Y., Rakovsky A., Mendelson E., Mor O. (2015). Evaluation of Xpert HIV-1 Qual assay for resolution of HIV-1 infection in samples with negative or indeterminate Geenius HIV-1/2 results. J. Clin. Virol..

[36] Wittawatmongkol O., Vanprapar N., Chearskul P., Phongsamart W., Prasitsuebsai W., Sutthent R., Chokephaibulkit K. (2010). Boosted p24 antigen assay for early diagnosis of perinatal HIV infection. J. Med. Assoc. Thail..

[37] Boyle D.S., Lehman D.A., Lillis L., Peterson D., Singhal M., Armes N., Parker M., Piepenburg O., Overbaugh J. (2013). Rapid detection of HIV-1 proviral DNA for early infant diagnosis using recombinase polymerase amplification. MBio.

[38] Fearon M. (2005). The laboratory diagnosis of HIV infections. Can. J. Infect. Dis. Med. Microbiol..

[39] Moorehouse M., Conradie F., Venter F. (2016). What is the role of CD4 count in a large public health antiretroviral programme?. S. Afr. J. HIV Med..

[40] Masur H., Ognibene F.P., Yarchoan R., Shelhamer J.H., Baird B.F., Travis W., Suffrendini A.F., Deyton L., Kovacs J.A., Falloon J. (1989). HIV targets CD4+ T cells, which are the most crucial cells of the immune system as the immune system uses these cells to fight infection. J. Ann. Intern. Med..

[41] Boyle D.S., Hawkins K.R., Steele M.S., Singhal M., Cheng X. (2012). Emerging technologies for point of care CD4 T-lymphocyte counting. Trends Biotechnol..

[42] Glynn M.T., Kinahan D.J., Ducree J. (2013). CD4 counting technologies for HIV therapy monitoring in resource poor settings-state of the art and emerging microtechnologies. Lab Chip.

[43] Pointcare 2010 8 Minutes to Better HIV/AIDS Patient Care. http://www.pointcare.net/docs/pointcare_now_brochure_eng.pdf.

[44] Bergeron M., Daneau G., Ding T., Sitoe N.E., Westerman L.E., Stokx J., Jani I.V., Coetzee L.M., Scott L., De Weggheleire A. (2012). Performance of the PointCare Now system for CD4 counting in HIV patients based on five independent evaluations. PLoS ONE.

[45] Shott J.P., Galiwango R.M., Reynolds S.J. (2012). A Quality Management Approach to Implementing Point-of-Care Technologies for HIV Diagnosis and Monitoring in Sub-Saharan Africa. J. Trop. Med..

[46] Wade D., Diaw P.A., Daneau G., Diallo A.A., Mboup S., Dieye T.N., Kestens L. (2015). Laboratory and field evaluation of the Partec CyFlow MiniPOC for absolute and relative CD4 T-cell enumeration. PLoS ONE.

[47] Wang S., Liffson M.A., Inci F., Liang L., Sheng Y., Demirci U. (2016). Advance in addressing technical challenges of point of care diagnostics in resource limited settings. Expert Rev. Mol. Diagn..

[48] Cheng X., Liu Y.S., Irimia D., Demirci L.J., Yang L., Zamir W., Rodrguez R., Toner M., Bashir R. (2007). Cell detection and counting through cell lysate impedance spectroscopy in microfluidic devices. Lab Chip.

[49] Logan C., Givens M., Ives J.T., Delaney M., Lochhead M.J., Schooley R.T., Benson C.A. (2013). Performance evaluation of the MBio diagnostics point of care CD4 counter. J. Immunl. Methods.

[50] World Health Organization (2014). Who Prequalification of Diagnostic Programme: Public Report.

[51] Bwana P., Vojnov L., Adhiambo M., Akinyi C., Mwende J., Prescott M., Mwau M. (2016). The BD FACSPresto point of care CD4 test accurately enumerates CD4+ T cell sounts. PLoS ONE.

[52] Chin C.D., Linder V., Sia S.K. (2012). Commercialization of microfluidic point of care diagnostic devices. Lab Chip.

[53] World Health Organization (2011). WHO Prequalification of Diagnostics Programme: Public Report, Pima CD4 Test. http://www.who.int/diagnostics_laboratory/evaluations/111208_0099_032_00_public_report_v2.pdf.

[54] Kasama S., Nattawat O., Charin T., Korakot P., Boonrat T., Kovit P. (2011). Performance evaluation of the Alere PIMA CD4 test for monitoring HIV-infected individuals in resource constrained setting. J. Acquir. Immune Defic. Syndr..

[55] Jung W., Han J., Choi J., Ahn C. (2015). Point of care testing (POCT) diagnostic systems using microfluidic lab on a chip technologies. Microelectron. Eng..

[56] Vashist S.K., Luppa P.B., Yeo L.Y., Ozcan A., Luong J.H.T. (2015). Emerging technologies for next-generation point of care testing. Trends Biotechnol..

[57] Drain P.K., Rousseau C. (2017). Point of care diagnostics: Extending the laboraoty network to reach the last lime. Curr. Opin. HIV AIDS.

[58] Nayak S., Blumenfeld N.R., Laksanasopin T., Sia S.K. (2017). Point of care diagnostics: Recent developments in a connected age. Anal. Chem..

